# 

**Published:** 1902-07-19

**Authors:** 


### NOTICE TO CORRESPONDENTS.

All MSS., Letters, Books for Review, and other matters intended for the Editor should be addressed The Editor, "The Hospital," The Hospital Building, 28 & 29 Southampton Street, Strand, W.C.
The Editor cannot undertake to return rejected MS., even when accompanied by stamped directed envelope.

**Notice to Advertisers and Subscribers.**

All Advertisements, Orders for Copies of The Hospital, and Business Communications should be addressed to **THE MANAGER, (Not to the Editor)**, The Hospital, 28 & 29 Southampton Street, Strand, London, W.C.
The Cost of Subscription, post free, is as follows.—Per week, 2½d.; per quarter, 2s. 8d.; per half-year, 5s. 3d.; per annum, 10s. 6d. Foreign:—Per annum, 15s. 2d., payable, in all cases, in advance.

**NOTICE.—Vol. XXXI. of "The Hospital" (October 1901, to March 1902), in handsome cloth binding is now on sale at the office of this Journal, price, 7s. 6d. Cases for binding the Numbers contained in this Volume 1s. 6d. Index to Vol. XXXI., 2½d., post free.**
**The Monthly Part for July is now ready. Price 6d., by post 8d.**

### Scraps and Gleanings.

Since the Birmingham Hospital Sunday movement was started over £203,000 has been handed over to the medical institutions and charities of Birmingham.

Upwards of £574 was collected this year in Hull on Hospital Saturday, so that this year's collection very nearly comes up to the record collection of last year, namely, £590.

At a recent special general meeting of the Dover Hospital, a presentation was made to Mr. E. Elwin in recognition of his services as honorary secretary for 33 years. The presentation was made by the Mayor.

Madame Bernhardt and other distinguished artists have promised their services in connection with the entertainment on behalf of the League of Mercy which Catharine Duchess of Westminster and Lady Grosvenor are organising.

At a recent meeting of the Oakwell (Batley) Joint Hospital Board it was agreed to erect a hospital for infectious diseases at the cost of about £10,000, including the expense of the site. Provision will be made for 26 patients, and the whole work is to be done in a thoroughly up-to-date style.

Mr. Arthur Appleby, J.P., has given £1,000 towards the cost of carrying out certain much-needed extensions at the Accrington Victoria Hospital. An additional gift of £500, making £1,000 in all, has also been received by this institution from the trustees of the late Alderman Whittaker.

During last year the receipts of the general fund of the Victoria Cottage Hospital, Romford, amounted to £475, and the expenditure was £489. The committee anxiously appeal for an increase of £300 in their annual subscriptions, which at present amount to only £190, whereas £500 is required from this source.

Great preparations are being made to ensure the success of the bazaar which is to be held on August 13 and 14, in aid of the Dover Hospital. The Countess Grosvenor is one of the stall-holders, the list of whom includes the names of many well-known ladies. It is confidently hoped that it may prove a great financial success, as more than £9,000 is required to meet the first two contracts for the additions and alterations at the Dover Hospital, and the committee have at present only £6,000 in hand towards this sum.

### THE STUDENT OF MEDICINE.

### APPOINTMENTS.

**Thomas Bell**, M.D.Glasg., M.S., appointed Certifying Factory Surgeon for the Shepshed District of Leicestershire, and Medical Officer for the Shepshed District of the Loughborough Union. **W. G. H. Bradford**, B.A., M.B., B.C.Cantab., appointed Medical Officer for the Brenchley District, and Public Vaccinator for the Sixth and Seventh Districts of the Tonbridge Union. **Gilbert Edward Butler**, M.R.C.S.Eng., L.R.C.P.Lond., appointed Medical Officer for Zeehan, and Health Officer for Montagu, Tasmania. **F. W. J. Coaker**, F.R.C.S.Eng., L.R.C.P.Lond., appointed District Medical Officer of the Bromsgrove Union. **W. E. Llewelyn Davies**, M.R.C.S.Eng., L.R.C.P.Lond., appointed Assistant Medical Officer to the Mile End Old Town Infirmary. **G. Downes**, M.R.C.S.Eng., L.R.C.P.Lond., appointed District Medical Officer of the Royston Union. **B. J. Dudley**, B.A.Cantab., M.R.C.S.Eng., L.R.C.P., appointed Second Assistant Medical Officer to the Greenwich Union Infirmary. **J. E. Dunn**, L.R.C.P.Edin., M.R.C.S.Eng., appointed District Medical Officer of the Preston Union. **Chas. Henry Evers**, M.R.C.S.Eng., L.S.A., appointed Medical Officer and Public Vaccinator for the No. 6 District of the Morpeth Union. **C. H. Falvey**, L.R.C.P., L.R.C.S.Irel., appointed Certifying Factory Surgeon for the Ardara District of Donegal. **Basil Faulds**, M.R.C.S.Eng., L.R.C.P.Lond., appointed Visiting Medical Officer to the Carrington Convalescent Hospital, Camden, New South Wales. **George E. Froggatt**, M.B., B.S.Durh., appointed Assistant Medical Officer to the Bethnal Green Infirmary. **W. F. L. Austen Holcroft**, M.B., B.Ch.Edin., appointed Medical Officer and Public Vaccinator for the Second and Fifth Districts of the South Molton Union, Devon. **T. C. Lawson**, M.R.C.S.Eng., appointed District Medical Officer of the Clun Union. **R. Lee**, M.B., B.Ch.R.U.I., appointed Assistant Medical Officer to the Cardiff Union. **A. Mercer**, M.R.C.S., L.R.C.P.Lond., appointed District Medical Officer of the Bolton Union. **W. E. Morgan**, M.R.C.S., L.R.C.P.Lond., appointed Medical Officer of the Camberwell Union. **A. V. Peatling**, B.A., M.B., B.C.Cantab., appointed Honorary Medical Officer to the Carshalton Cottage Hospital. **Frederick Maurice Purchas**, M.B., M.S.Edin., appointed Health Officer for the Port of Kaipara. **John Stewart**, L.K.Q.C.P., L.R.C.S.Irel., appointed Medical Officer of Health at Hamilton, Tasmania. **W. M. Thomas**, M.R.C.S., L.R.C.P., appointed District Medical Officer of the Liskeard Union. **W. Ernest Thomson**, M.A., M.D., F.F.P.S.Glasg., appointed Ophthalmic Physician to the Glasgow Maternity Hospital. **J. Waters**, L.S.A., appointed District and Workhouse Medical Officer of the Cosford Union. **G. C. Wilkin**, M.R.C.S.Eng., L.R.C.P.Lond., appointed District Medical Officer of the Towcester Union. **R. Winterbotham**, M.R.C.S., L.R.C.P.Lond., appointed District Medical Officer of the Brixworth Union.

### "The Hospital" Institutional Library.

"Hospitals and Asylums of the World." 4 Vols., with a Portfolio of Plans, complete £12 12s.

"Burdett's Hospitals and Charities, 1902." 5s.

"Cottage Hospitals, General, Fever, and Convalescent." 10s. 6d.

"Hospital Expenditure: The Commissariat." 2s. 6d.

"A Legal Handbook for the Use of Hospital Authorities." 2s. 6d.

"The Cottage Hospital Case Book or Register of Patients." 10s. and 12s. 6d.

"The Furnishing and Appliances of a Cottage Hospital." 6d.

All these are published by the Scientific Press, Ltd., and may be obtained through any bookseller, or direct from the publishers, 28 and 29 Southampton Street, Strand, London, W.C.

